# Critical reappraisal of neoadjuvant concurrent chemoradiotherapy for treatment of locally advanced colon cancer

**DOI:** 10.1371/journal.pone.0259460

**Published:** 2021-11-02

**Authors:** Yen-Cheng Chen, Hsiang-Lin Tsai, Ching-Chun Li, Ching-Wen Huang, Tsung-Kun Chang, Wei-Chih Su, Po-Jung Chen, Tzu-Chieh Yin, Chun-Ming Huang, Jaw-Yuan Wang

**Affiliations:** 1 Division of Colorectal Surgery, Department of Surgery, Kaohsiung Medical University Hospital, Kaohsiung Medical University, Kaohsiung, Taiwan; 2 Graduate Institute of Clinical Medicine, College of Medicine; Kaohsiung Medical University, Kaohsiung, Taiwan; 3 Faculty of Medicine, Department of Surgery, College of Medicine, Kaohsiung Medical University, Kaohsiung, Taiwan; 4 Division of General and Digestive Surgery, Department of Surgery, Kaohsiung Medical University Hospital, Kaohsiung Medical University, Kaohsiung, Taiwan; 5 Department of Surgery, Kaohsiung Municipal Tatung Hospital, Kaohsiung Medical University, Kaohsiung, Taiwan; 6 Department of Radiation Oncology, Kaohsiung Municipal Ta-Tung Hospital, Kaohsiung Medical University, Kaohsiung, Taiwan; 7 Department of Radiation Oncology, Kaohsiung Medical University Hospital, Kaohsiung Medical University, Kaohsiung, Taiwan; 8 Faculty of Medicine, Department of Radiation Oncology, College of Medicine, Kaohsiung Medical University, Kaohsiung, Taiwan; 9 Graduate Institute of Medicine, College of Medicine, Kaohsiung Medical University, Kaohsiung, Taiwan; 10 Center for Cancer Research, Kaohsiung Medical University, Kaohsiung, Taiwan; 11 Center for Liquid Biopsy and Cohort Research, Kaohsiung Medical University, Kaohsiung, Taiwan; 12 Pingtung Hospital, Ministry of Health and Welfare, Pingtung, Taiwan; Emory University School of Medicine, UNITED STATES

## Abstract

**Background:**

Locally advanced colon cancer (LACC) is associated with surgical challenges during R0 resection, increased postoperative complications, and unfavorable treatment outcomes. Neoadjuvant concurrent chemoradiotherapy followed by surgical resection is an effective treatment strategy that can increase the complete surgical resection rate and improve the patient survival rate. This study investigated the efficacy and toxicity of concurrent chemoradiotherapy in patients with LACC as well as the prognosis and long-term clinical outcomes of these patients.

**Materials:**

From January 2012 to July 2020, we retrospectively reviewed the real-world data of 75 patients with LACC who received neoadjuvant concurrent chemoradiotherapy. The chemotherapy regimen consisted of folinic acid, 5-fluorouracil, and oxaliplatin (FOLFOX). The following data were obtained from medical records: patients’ characteristics, pathologic results, toxicity, and long-term oncologic outcome.

**Results:**

Of the 75 patients, 13 (17.3%) had pathologic complete responses. Hematologic adverse effects were the most common (grade 1 anemia: 80.0% and leukopenia: 82.7%). Conversely, grade 2 or 3 adverse effects were relatively uncommon (<10%). Pathologic N downstaging, ypT0, and pathologic complete responses were significant prognostic factors for patient survival. Multivariate analysis revealed that pathologic N downstaging was an independent predictor of patients’ overall survival (*P* = 0.019). The estimated 5-year overall and disease-free survival rates were 68.6% and 50.6%, and the medians of overall and disease-free survival periods were 72.3 and 58.7 months, respectively. Moreover, patients with pathologic complete responses had improved overall survival (*P* = 0.039) and an improved local recurrence control rate (*P* = 0.042) but an unfavorable distant metastasis control rate (*P* = 0.666) in the long-term follow-up.

**Conclusion:**

The long-term oncologic outcome of patients with LACC following concurrent chemoradiotherapy is acceptable, and the adverse effects seem to be tolerable. Pathologic N downstaging was an independent prognostic factor for patients’ overall survival. However, a large prospective, randomized control study is required to confirm the current results.

## Introduction

Colorectal cancer (CRC) is a worldwide malignant disease with a high prevalence, and nearly 9% of cancers are derived from CRC [1,2]. It is the third most common cancer in the world, with a rapidly increasing prevalence [2,3]. In Asia, CRC increases the mortality rate and causes major health problems [1,4]. In Taiwan, CRC is now the most common cancer according to the data from Ministry of Health and Welfare of Taiwan [5]. Therefore, the treatment strategy for CRC has become an emergent topic. For colon cancer, surgical resection with free margin is currently the most recommended curative therapy [6–8]. According to National Comprehensive Cancer Network (NCCN) guidelines before 2015, for non-metastatic, resectable colon cancer, surgical resection is the most recommended treatment [9,10]. However, approximately 15% of colon cancers are locally advanced colon cancer (LACC) without distant metastasis (DM), which involves invasion to other organs or lymph node metastasis encasing the root of the main feeding artery [3,6,11,12]. In these cancers, curative surgical resection followed by adjuvant chemotherapy may be a treatment option [7,8,12]. However, achieving R0 resection can be challenging, and this type of resection is associated with high morbidity rates [3,11], with an incidence of up to 20% for postoperative complications [6]. As expected, the treatment outcome of LACC is unsatisfactory. The 5-year overall survival (OS) rate is low and ranges from 13% to 73% depending on the invasion depth and lymph node metastasis [7,8,13]. Therefore, recently, neoadjuvant concurrent chemoradiotherapy (CCRT) followed by surgical resection has been proposed to treat LACC [14,15].

Neoadjuvant CCRT has been widely administered for the treatment of locally advanced rectal cancer (LARC) compared with LACC [6–8,11,12,16–18]. Tumor downstaging can increase the success rate of tumor resection and prevent local recurrence (LR) [6,12,17–20]. Another possible advantage of neoadjuvant CCRT is the eradication of occult systemic micro-metastasis, which might decrease cancer DM [8,12,19]. With tumor regression and shrinkage, surgical resection can be less challenging, resulting in fewer postoperative complications [7,8,18,19]. Moreover, patients are less likely to delay adjuvant therapy due to postoperative complication-related infection or malnutrition [12]. Because of the aforementioned factors, neoadjuvant CCRT has recently been shown to have survival benefits in LARC treatment [6,12,13,17,18].

Because the most common pathologic diagnosis of CRC is adenocarcinoma [6], several studies have introduced neoadjuvant CCRT in LACC treatment and have shown promising results [6,8,11,12]. Almost all studies have indicated that neoadjuvant CCRT is a feasible and safe treatment strategy and is effective for treating LACC, particularly unresectable tumors [6–8,11–13]. However, few findings are available regarding the long-term oncological outcomes of neoadjuvant CCRT. One study indicated a long-term survival benefit of neoadjuvant CCRT [6], whereas another study presented similar survival rates between neoadjuvant and adjuvant CCRT [12]. The data of long-term follow-up of patients treated with neoadjuvant or adjuvant CCRT are still lacking. In an earlier study, we presented real-world evidence of patients with LACC with neoadjuvant CCRT and subsequent surgical resection [11]. Herein, we present our long-term follow-up data of 75 patients with LACC after neoadjuvant CCRT and curative-intent surgical resection.

## Materials and methods

### Patients

Adenocarcinoma diagnosis in patients with LACC was confirmed through histopathologic findings. Treatment strategies, such as surgical resection, chemotherapy, or chemoradiotherapy, were discussed in multidisciplinary team (MDT) cancer conferences. Colorectal surgeon, radiologist, radiation oncologist and pathologist were included in the treatment team. Following shared decision-making principle, we discussed with patients and their family to make an optimal treatment program. LACC was defined based on computed tomography (CT) findings, which included (1) T3 tumor with extramural extension of >5 mm, (2) clinical T4 stage tumor, and (3) extensive lymph node metastasis at the root of the main feeding artery. On the basis of image findings from CT, we defined the clinical stage of colon cancer and possibility of complete tumor resection. From January 2012 to July 2020, 75 patients with LACC were considered unsuitable for upfront surgical resection and received neoadjuvant CCRT after decision-making at MDT conferences. Surgical resection was performed based on the tumor response. All patients met the criterion of Eastern Cooperative Oncology Group score of 0–2 without DM. Pretreatment evaluation entailed complete medical history review and physical examination, colonoscopy, tumor biopsy, chest radiography, abdominal and pelvic CT scan with or without magnetic resonance imaging, serum carcinoembryonic antigen (CEA) level assessment, and routine laboratory tests. Patients with a history of synchronous malignancies other than non-melanoma skin cancer or major medical comorbidities that may affect treatment compliance were excluded. We reviewed the medical records of patients and collected their data, including demographic characteristics, image features, biochemistry examinations, treatment efficacy and toxicity, histopathological features, and oncologic outcomes. The present study was approved by the institutional ethics committee of our hospital (KMUHIRB-E(I)-20200036).

### Preoperative treatment

The clinical tumor stage after neoadjuvant CCRT was determined through an abdominal CT scan, and clinical T and N stages were assessed through preoperative evaluation. Concurrent radiotherapy and chemotherapy were applied for all patients. We used mFOLFOX6 as the chemotherapy regimen. mFOLFOX6 consisted of oxaliplatin (85 mg/m^2^, as a 4-hour intravenous [IV] infusion) on day 1 followed by leucovorin (200 mg/m^2^, as a 2-hour IV infusion) and fluorouracil (2800 mg/m^2^ as a 46-hour IV infusion); this regimen was repeated biweekly. Before radiotherapy, all patients underwent a planning CT scan for tumor localization. According to the diagnostic CT scan, the macroscopic tumor and enlarged lymph nodes were defined as gross tumor volume. Gross tumor volume plus a 15- to 20-mm margin was defined as clinical target volume. For left side tumor, we applied 45 Gy in 25 fractions on lymphatic drainage around inferior mesenteric vein and pelvic area. A simultaneous boost of 50 Gy was delivered to primary tumor. For right side tumor, we applied 45 Gy in 25 fractions on possible lymph nodes over superior mesenteric vein area. A simultaneous boost of 50 Gy was delivered to primary tumor. Both Three-dimensional conformal radiation therapy and intensity‑modulated radiotherapy were used depending on patient’s clinical condition. Tomotherapy (TomoTherapy Hi.Art System®) was used to perform image-guided radiotherapy and intensity modulated radiotherapy. Linear accelerator (Elekta Synergy®) was used for volumetric modulated arc therapy and image-guided radiotherapy. 3 dimensions conformal radiotherapy was conducted by using a four-filed box technique. The volume of a small bowel receiving >50 Gy was limited to <1 cc, and the maximal dose to the spinal cord was restricted to <45 Gy.

Chemotherapy and radiotherapy were initiated at the same time. After radiotherapy completion, we kept chemotherapy biweekly until 2~3 weeks before surgery. The median time interval from completion of radiotherapy to surgical resection was 7 weeks (range from 6 to 8 weeks). Generally, the patients received a median of 6 cycles of neoadjuvant chemotherapy (range from 6 to 7 cycles). During this period, we measured the serum CEA level at each cycle of chemotherapy. We performed an enhanced abdominal CT scan after every six cycles of chemotherapy or if patients had two consecutive abnormal CEA levels. Using CT, we evaluated the tumor response and established a surgical plan.

### Surgery and pathology review

More than 6 weeks after the completion of radiotherapy, patients underwent elective surgery. During this period, we continued to administer chemotherapy biweekly until surgery. Typical radical resection of tumors and lymph nodes was performed. The pathologic tumor stage was confirmed based on tumor invasion (ypT) and nodal metastasis (ypN). The statuses of the circumferential, proximal, and distal resection margins were identified. A circumferential resection margin (CRM) of <1 mm was defined as a positive CRM. Other pathologic features included histological grade, tumor regression grade, perineural invasion, and lymphovascular invasion. We used the American Joint Committee on Cancer system to assess cancer responses: grade 0, no residual cancer cells; grade 1, a single cell or a small group of cancer cells (major regression); grade 2, residual cancer with a desmoplastic response (moderate regression); and grade 3, minimal evidence of tumor responses. A pathologic complete response (pCR) was defined as the absence of viable cancer cells in the primary tumor and lymph node specimens (ypT0N0).

### Postoperative adjuvant chemotherapy

After the resection of the primary tumor, risk factors for pathologic findings were reviewed. Furthermore, the pathologic stage was recorded to determine the further adjuvant chemotherapy regimen. For patients with pathologic T-positive tumors, lymph node metastasis, positive resection, or circumferential margin, we administered adjuvant chemotherapy with mFOLFOX6 up to 12 cycles and repeated it every 2 weeks. For patients with pCR, we applied fluoropyrimidine-based chemotherapy for up to 6 months, with close follow-up. If patients developed LR or DM, we applied folinic acid, fluorouracil, and irinotecan or folinic acid, 5-fluorouracil, and oxaliplatin (FOLFOX) regimen with target therapy according to the *RAS* gene status.

### Toxicity evaluation and follow-up

In each cycle of neoadjuvant and adjuvant chemotherapy, adverse effects (AEs) were assessed using Common Terminology Criteria for Adverse Events, version 4.0 [21].

### Statistical analysis

Descriptive statistics are presented as proportions and means. We used the SPSS software package (version 20, International Business Machines Corporation Inc., Armonk, New York, USA) for statistical analysis of all data. All patients were followed up until their death or July 31, 2020. The chi-squared test and logistic regression were used to compare categorical data. *P* < 0.05 was defined as statistical significance. OS was defined as the time from treatment onset to death date from any cause or to the date of the final follow-up. Disease-free survival (DFS) was defined as the time from treatment onset to the date of diagnosis of LR or distant metastasis or the date of the final follow-up. DFS and OS were evaluated using the Kaplan–Meier method, and the log-rank test was used to compare time-to-event distributions.

## Results

### Patient characteristics

Among 75 patients, 45 were men (60.0%), and 30 were women (40.0%). In total, 72 resected advanced colon cancers were included in this study. Six patients (8.0%) had clinically unresectable tumors and could not undergo surgical resection due to poor tumor responses and their health condition. One patient had three synchronous advanced colon cancers, and one patient had two synchronous advanced colon cancers. The median age of patients was 65 years (range, 41–89 years). Half (51.3%) and one-third (29.5%) of the tumors were located in the sigmoid colon and ascending colon, respectively. The proportions of right- and left-side tumors were 45.2% and 54.8%, respectively. Regarding tumor depth, 38 patients had tumors staged as T3 (48.7%), and 40 patients had T4 tumors (51.3%). Nearly all patients presented clinical lymph node metastasis with clinical stage III (98.7%), and only one patient presented clinical stage II as T4bN0M0. The median CEA level before treatment was 6.14 ng/mL, ranging from 0.6 to 649 mg/mL. The CEA level was >5 ng/mL in about half (56%) of the patients. After treatment, the CEA level was <2 ng/mL in 32 patients (42.7%). 43 patients (57.3%) received diversion colostomy or ileostomy as bridge therapy for bowel decompression. Table 1 lists the patient and treatment characteristics.

**Table 1:** Summary and characteristics of patients (patients, N = 75; resected tumor, N = 72)^a^.

Characteristic	
**Age (years, median) (range)**	65 (41–89)
**Gender**	
Male	45 (60.0%)
Female	30 (40.0%)
**BMI kg/m**^**2**^ **(mean) (range)**^b^	23.2 (15.4–35.5)
**Location**	
Cecum	8 (10.3%)
Ascending colon	23 (29.5%)
Transverse colon	4 (5.1%)
Descending colon	3 (3.8%)
Sigmoid colon	40 (51.3%)
**Location (exclude synchronous tumor)**	
Right colon	33 (45.2%)
Left colon	40 (54.8%)
**Clinical tumor depth**	
T3	38 (48.7%)
T4a	24 (30.8%)
T4b	16 (20.5%)
**Clinical lymph node metastasis**	
N0	1 (1.3%)
N1	39 (50.0%)
N2	38 (48.7%)
**7th Edition of the AJCC staging** ^c^	
IIC	1 (1.3%)
IIIB	46 (59.0%)
IIIC	31 (39.7%)
**Pretreatment CEA (ng/mL)(median)** ^d^	4.4 (0.6–649)
**CEA≦5 (ng/mL)**	
Yes	33 (44%)
No	42 (56%)
**After treatment CEA<2 (ng/mL)**	
Yes	32 (42.7%)
No	43 (57.3%)
**Ileosotmy/colostomy prior to therapy**	
Yes	43 (57.3%)
No	32 (42.7%)
**Failed to receive surgical resection**	
Yes	6 (8.0%)
No	69 (92.0%)

^a^Among 75 patients, six failed to receive surgical resection, one patient had three synchronous advanced colon cancer, and one patient had two synchronous advanced colon cancer

^b^BMI, body mass index

^c^AJCC, American Joint Commission on Cancer

^d^CEA, carcinoembryonic antigen.

### Pathologic response

In total, 72 resected tumors were analyzed through histopathological examination. Of these, 13 specimens had no visible cancer cells at the primary site (pT0), 39 specimens presented serosa invasion under microscopy (pT3), and 9 specimens showed tumor invasion out of the colon (pT4a and pT4b). In total, no pathologic lymph node metastasis (pN0) occurred in 57 specimens (79.2%); moreover, 13 (17.3%) patients presented pCR with no viable cancer cells in the specimens (In resected tumor (N = 72), the pCR rate was 18.1%. In total patients (N = 75), the pCR rate was 17.3%. We used 17.3% as our pCR rate in the following paragraph). Both proximal and distal resection margins were free of cancer cells, but three specimens (4.2%) revealed positive CRMs. On microscopic examination, 13 specimens (18.1%) showed lymphovascular invasion, and 12 (16.7%) showed perineural invasion. Most tumors were moderately differentiated (83.3%), and 5.6% and 11.1% of the tumors were well and poorly differentiated, respectively.

Considering the pathologic T stage, 41 specimens (56.9%) showed tumor downstaging to ypT0-2, and 30 specimens (41.7%) showed a stable stage. Most specimens (84.7%) presented pathologic N downstaging. Considering the overall clinical T and N stages, 58 specimens (80.6%) showed downstaging, and 11 specimens (15.3%) had a stable stage. However, three specimens presented progressed stages. Table 2 summarizes the pathologic evaluation results of primary tumors after neoadjuvant CCRT.

**Table 2:** Pathological results and tumor response of neoadjuvant treatment (resected tumor, N = 72).

	No. (%)
ypT	
0	13 (18.1)
1	1 (1.2)
2	10 (13.9)
3	39 (54.2)
4a	5 (6.9)
4b	4 (5.6)
**ypN**	
0	57 (79.2)
1	12 (16.7)
2	3 (4.2)
**Pathologic complete response**	
Yes	13 (18.1)*
No	59 (82.0)
**Circumferential resection margin (CRM)**	
Negative	69 (95.8)
Positive	3 (4.2)
**Lymphovascular invasion**	
Yes	13 (18.1)
No	59 (82.0)
**Perineural invasion**	
Yes	12 (16.7)
No	60 (83.3)
**Tumor differentiation**	
Well	4 (5.6)
Moderate	60 (83.3)
Poorly	8 (11.1)
**Pathologic T stage**	
Down staging	41 (56.9)
Stable	30 (41.7)
Progressive	1 (1.4)
**Pathologic N stage**	
Down staging	61 (84.7)
Stable	9 (12.5)
Progressive	2 (2.8)
**Pathologic TN stage**	
Down staging	58 (80.6)
Stable	11 (15.3)
Progressive	3 (4.2)

*In resected tumor (N = 72), the pCR rate was 18.1%. In total patients (N = 75), the pCR rate was 17.3%.

### Toxicity of CCRT

Among all the AEs of CCRT, hematologic AE was the most common side effect. In total, 60 (80.0%) and 62 (82.7%) patients developed leukopenia and anemia, respectively; however, most of the AEs were mild to moderate (grades 1 or 2). The incidence of grade 3 anemia and leukopenia was 6.7% and 2.7%, respectively. The incidence of grade 1, 2, and 3 thrombocytopenia was 69.3%, 10.7%, and 5.3%, respectively. Regarding nonhematologic toxicity, fatigue and nausea were the leading AEs. In total, 32 (42.7%) and 31 (41.3%) patients developed grade 1 fatigue and grade 1 nausea, respectively. Severe AEs were relatively uncommon, and the incidence of grade 3 nonhematologic AEs was <10%. Paresthesia is a side effect worth further investigation. In total, 23 (30.7%) patients had grade 1 paresthesia during CCRT. Table 3 presents the AEs that occurred during neoadjuvant CCRT.

**Table 3:** Toxicities during neoadjuvant treatment (patient, N = 75).

Toxicity	Grade 1		Grade 2		Grade 3	
	No.	%	No.	%	No.	%
**Fatigue**	32	42.7	1	1.3	0	0
**Hematologic**						
Anemia	62	82.7	46	61.3	5	6.7
Leukopenia	60	80.0	34	45.3	2	2.7
Thrombocytopenia	52	69.3	8	10.7	4	5.3
**Gastrointestinal**						
Nausea	31	41.3	6	8	1	1.3
Vomiting	7	9.3	5	6. 7	0	0
Diarrhea	18	20	6	8	2	2.7
**Paresthesia**	23	30.7	5	6.7	1	1.3
**Oral mucositis**	5	6.7	3	4	0	0
**Dermatitis**	9	12	1	1.3	0	0

### Prognostic factors for patient survival

A univariate analysis of the correlation of clinical features with patient survival revealed that sex, right- or left-side colon cancer, and the CEA level exerted no significant effect on patient survival. Considering pathologic features, no correlation was observed among vascular invasion, perineural invasion, histological differentiation, and patient OS. However, pathologic N downstaging (*P* = 0.029), ypT0 (*P* = 0.049), and pCR (*P* = 0.049) were significant prognostic factors for patient OS based on univariate analysis (Table 4). In the present study, patients with ypT0 tumors did not present lymph node metastasis. In multivariate logistic regression analysis, pathologic N downstaging was an independent predictor of patient OS (*P* = 0.019; odds ratio, 4.125; 95% confidence interval, 1.266–13.436). Both LR and DM were poor independent prognostic factors for patient OS (both *P* = 0.001, Table 4).

**Table 4:** Univariate and multivariate analysis of predictors of overall survival (patient, N = 75).

Parameters	Survival Yes (N = 50)*	No (N = 25)	Univariate analysis		Multivariate analysis	
			*P* value	Number (%)	Odds ratio (95% CI)	*P* value
**Gender (male/female)**	33/17	12/13	0.144			
**Tumor location (Right/Left)**	23(47.9)/25(52.1)	10(40.0)/15(60.0)	0.623	33 (45.2)	0.725(0.272–1.931)	0.520
Initial CEA<5 level (yes/no) **	25(50.0)/25(50.0)	8(32.0)/17(68.0)	0.217	33 (44.0)	0.471(0.172–1.288)	0.142
**CEA <2 after Tx (yes/no)**	25(50.0)/25(50.0)	7(28.0)/18(72.0)	0.086	32 (42.7)	0.389(0.138–1.094)	0.073
**Vascular invasion (yes/no)**	10(20.4)/39(79.6)	3(15.0)/17(85.0)	0.742	13 (18.8)	0.688(0.168–2.820)	0.604
**Perineural invasion (yes/no)**	9(18.4)/40(81,6)	2(10.0)/18(90.0)	0.490	11 (15.9)	0.494(0.097–2.521)	0.396
**Histology PD/WD+MD**	6(12.5)/42(87.5)	5(20.8)/ 19(79.2)	0.489	11 (15.2)	1.842(0.500–6.791)	0.359
pT downstage (yes/no) ***	26(52.0)/24(48.0)	11(44.0)/ 14(56.0)	0.809	40 (58.3)	0.851(0.324–2.324)	0.744
**pN downstage (yes/no)**	44(88.0)/6(12)	16(64)/9 (36)	0.029	60 (80.0)	4.125(1.266–13.436)	0.019
**pTN downstage (yes/no)**	39(78)/11(22.0)	16(64.0)/9(16.0)	0.268	55 (73.3)	1.994(0.694–5.732)	0.200
**ypN0 (yes/no)**	38(76.0)/12(24.0)	15(60.0)/10(40.0)	0.183	53 (70.6)	2.111(0.753–5.915)	0.155
**ypT0 (yes/no)**	12(24.0)/38(76.0)	1(4.0)/24 (96.0)	0.049	13 (17.3)	7.579 (0.925–62.079)	0.059
**pCR**^a^ **(yes/no)**	12(24.0)/38(76.0)	1(4.0)/24 (96.0)	0.049	13 (17.3)	7.579(0.925–62.079)	0.059
**LR**^b^ **(yes/no)**	4(8.0)/46(92.0)	11(44.0)/ 14(56.0)	<0.001	15 (20.0)	0.111(0.030–0.403)	0.001
**DM**^c^ **(yes/no)**	8(16.0)/42(84.0)	14(56.0)/11(44.0)	0.001	22 (29.3)	0.150(0.050–0.447)	0.001

*Six patients failed to receive surgical resection after neoadjuvant CCRT.

**Some CEA data missed during data collection.

***For patients with more than one tumor, we analyzed the tumor with the worst response. Among the patient with three synchronous tumors, two revealed tumor downstage, but one presented progression. We considered this patient as no downstage. Furthermore, the patient who failed to receive surgery was considered as no tumor downstage.

^a^pCR, pathologic complete response

^b^LR, local recurrence

^c^DM, distant metastasis; ypT0, pCR; CI, confidence interval; LN, lymph node; WD, well differentiated; MD, moderately differentiated; PD, poorly differentiated; A, adenocarcinoma; M, mucinous carcinoma; CEA, carcinoembryonic antigen; Tx, treatment.

### Survival and treatment outcome

**Fig 1:**
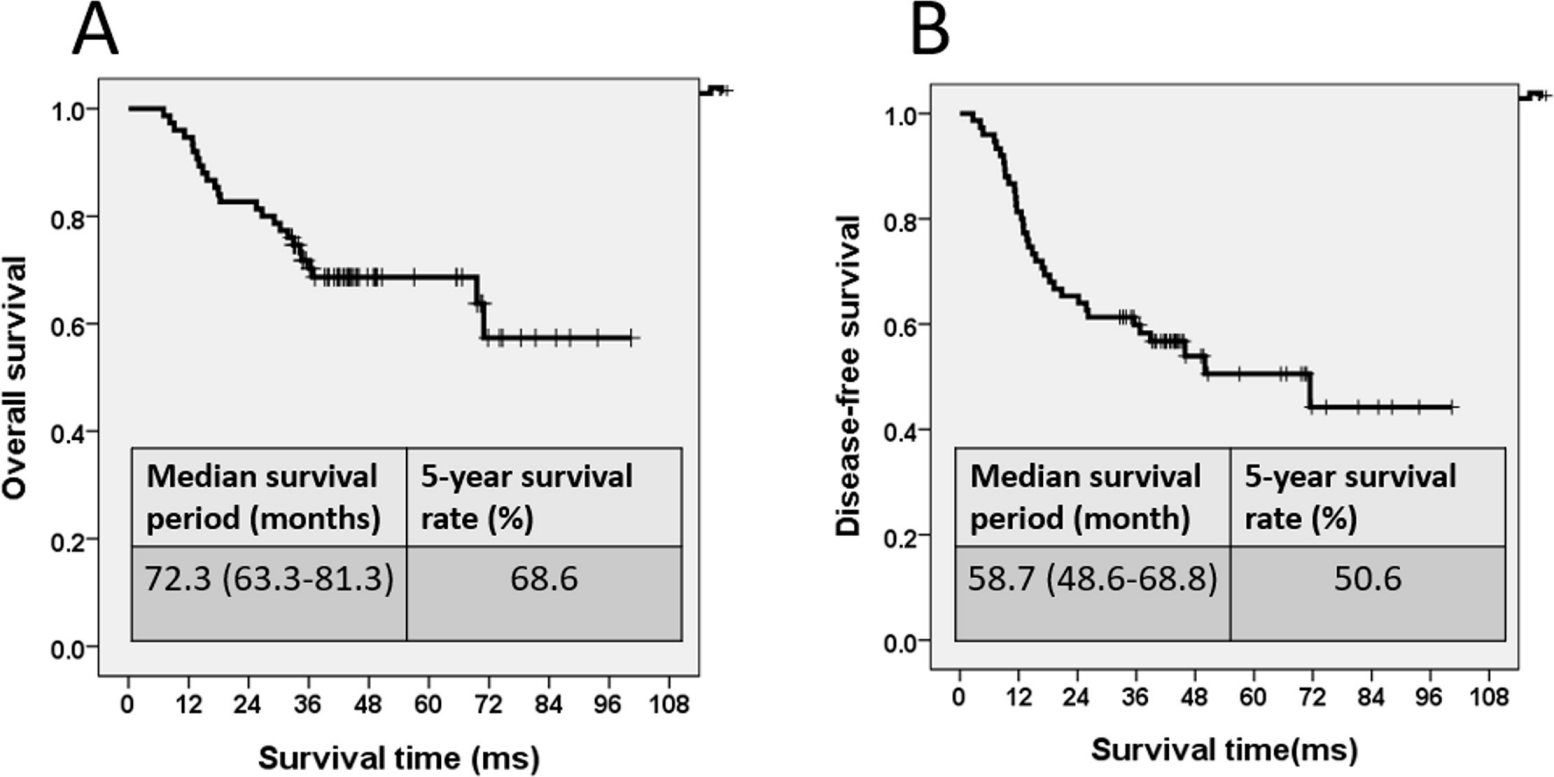
(A) Overall survival curve. (B) Disease-free survival curve.

The median follow-up period was 41.8 months (range: 7–100.4 months). The estimated 5-year OS rate was 68.6%, with a median OS period of 72.3 months (63.3–81.3 months, Fig 1A), and the estimated 5-year DFS rate was 50.6%, with a median DFS period of 58.7 months (48.6–68.8 months, Fig 1B). For patients with DM, the estimated 5-year OS rate was 37.8%, and the median survival period was 43.2 months (32.7–53.7 months). The distant metastatic sites included the liver, lung, peritoneum, para-aortic lymph nodes, and bones, and eight patients developed multiple metastasis simultaneously. For patients with LR, the estimated 5-year OS rate was 32.0%, and the median survival period was 42.0 months (29.8–54.1 months). Both DM and LR were poor prognostic factors with statistical significance. Patients with DM and LR presented with a shorter OS period than those without DM and LR (both *P* = 0.001).

**Fig 2:**
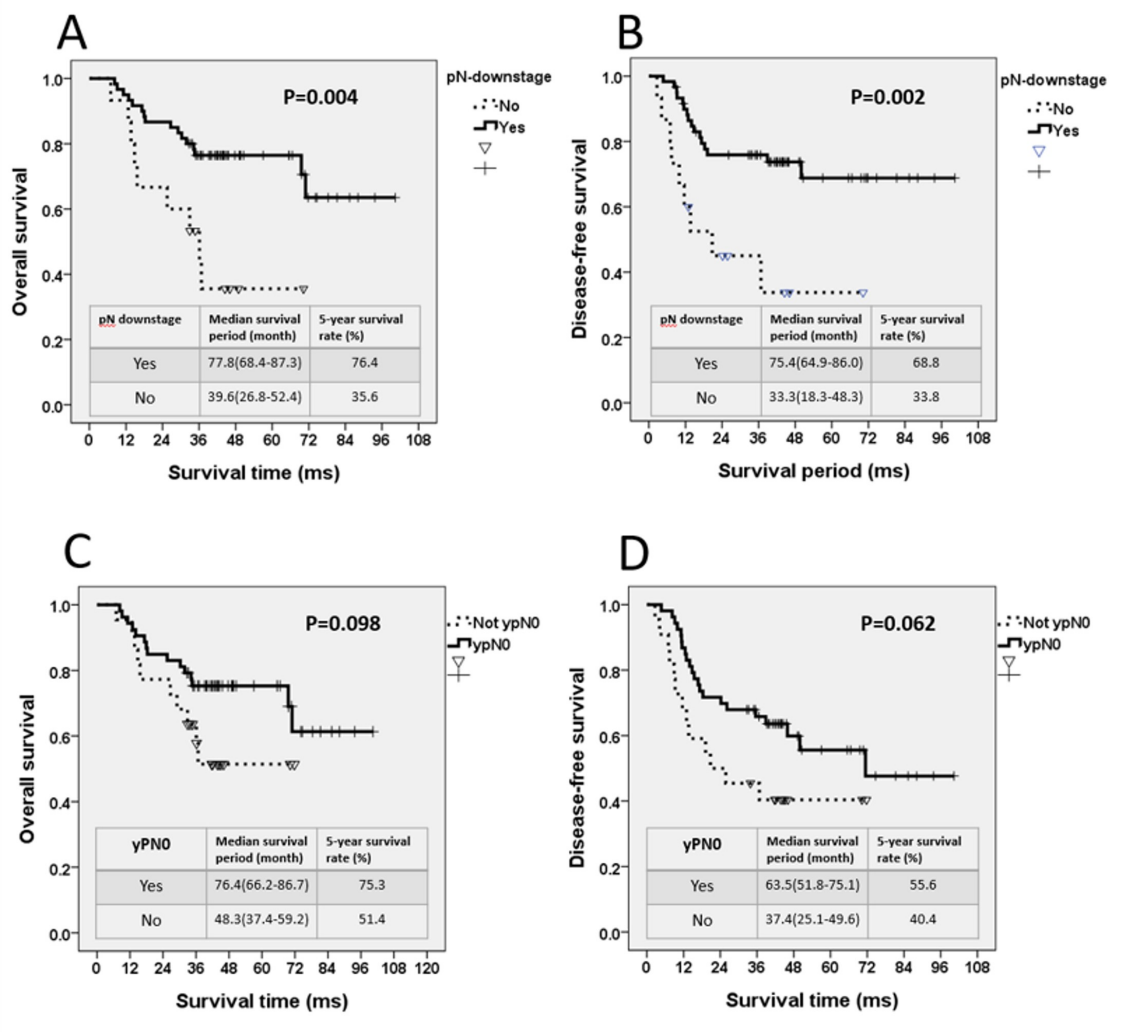
For patients with and without pN-downstage, (A) 5-year overall survival (OS) curve and (B) 5-year disease-free survival (DFS) curve. For patients with and without ypN0, (C) 5-year OS curve and (D) 5-year DFS curve.

In total, 60 patients (73.3%) showed pathologic N downstaging. For patients with pN-downstaging, the estimated 5-year OS and DFS rates were 76.4% and 68.8%, respectively. The median OS and DFS periods were 77.8 and 75.5 months, respectively. Furthermore, patients with pN-downstaging exhibited significantly improved OS (OS: 77.8 vs. 39.6 months, *P* = 0.004, Fig 2A) and DFS (DFS: 75.5 vs. 33.3 months, *P* = 0.002, Fig 2B).

In total, 53 patients (70.6%) had ypN0 tumors. In patients with ypN0 tumors, the estimated 5-year OS and DFS rates were 75.3% and 55.6%, respectively. The median OS and DFS periods were 76.5 and 71.4 months, respectively. Conversely, patients with pN0 tumors exhibited a trend of longer OS and DFS but without statistical significance (OS: 76.5 vs. 48.3 months, *P* = 0.098, Fig 2C; DFS: 71.4 vs. 20.8 months, *P* = 0.062, Fig 2D).

**Fig 3:**
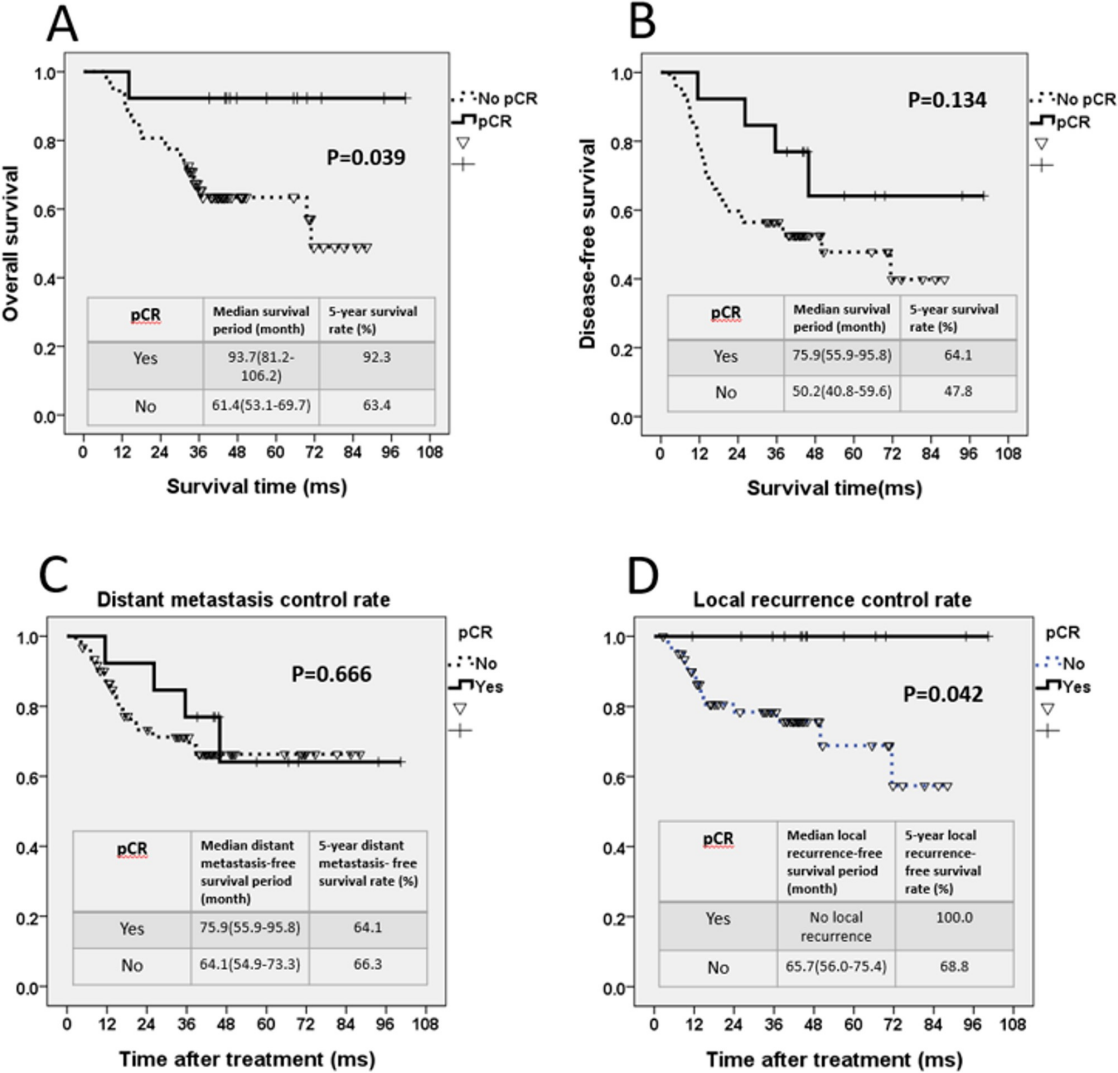
For patients with and without pCR, (A) the 5-year OS curve; (B) the 5-year DFS curve; (C) the distant metastasis control curve; and (D) the local recurrence control curve.

In the 13 (17.3%) patients who exhibited pCR, age and sex showed no obvious influence, and the proportions of stages IIIB and IIIC were not different. The estimated 5-year OS rate of patients showing pCR was 92.3% with a median OS period of 93.7 months, whereas the median OS period of patients without pCR was 61.4 months (*P* = 0.039, Fig 3A). The estimated 5-year DFS rate of patients showing pCR was 64.1% with a median DFS period of 75.9 months, whereas the median DFS period of patients without pCR was 50.2 months. Patients with pCR seemed to have a trend of longer DFS, but the difference was not statistically significant (*P* = 0.134, Fig 3B).

Among the 13 patients with pCR, four patients developed DM in the follow-up period. The metastatic sites were as follows: bone, multiple sites, liver, and peritoneum. However, the patient with T4b cancer didn’t sustain DM. In patients showing pCR, the median DM-free survival period was 75.9 months, and the estimated 5-year DM-free survival rate was 64.1%. However, for patients without pCR, the median DM-free survival period was 64.1 months, and the estimated 5-year DM-free survival rate was 66.3%. Patients with pCR exhibited no advantages in terms of DM control (*P* = 0.666, Fig 3C). For patients without pCR, the median LR-free survival period was 65.7 months, and the estimated 5-year LR-free survival rate was 68.8%. Conversely, patients with pCR exhibited no LR in the entire follow-up period (*P* = 0.042, Fig 3D).

### CEA level, tumor location, and treatment outcome

**Fig 4:**
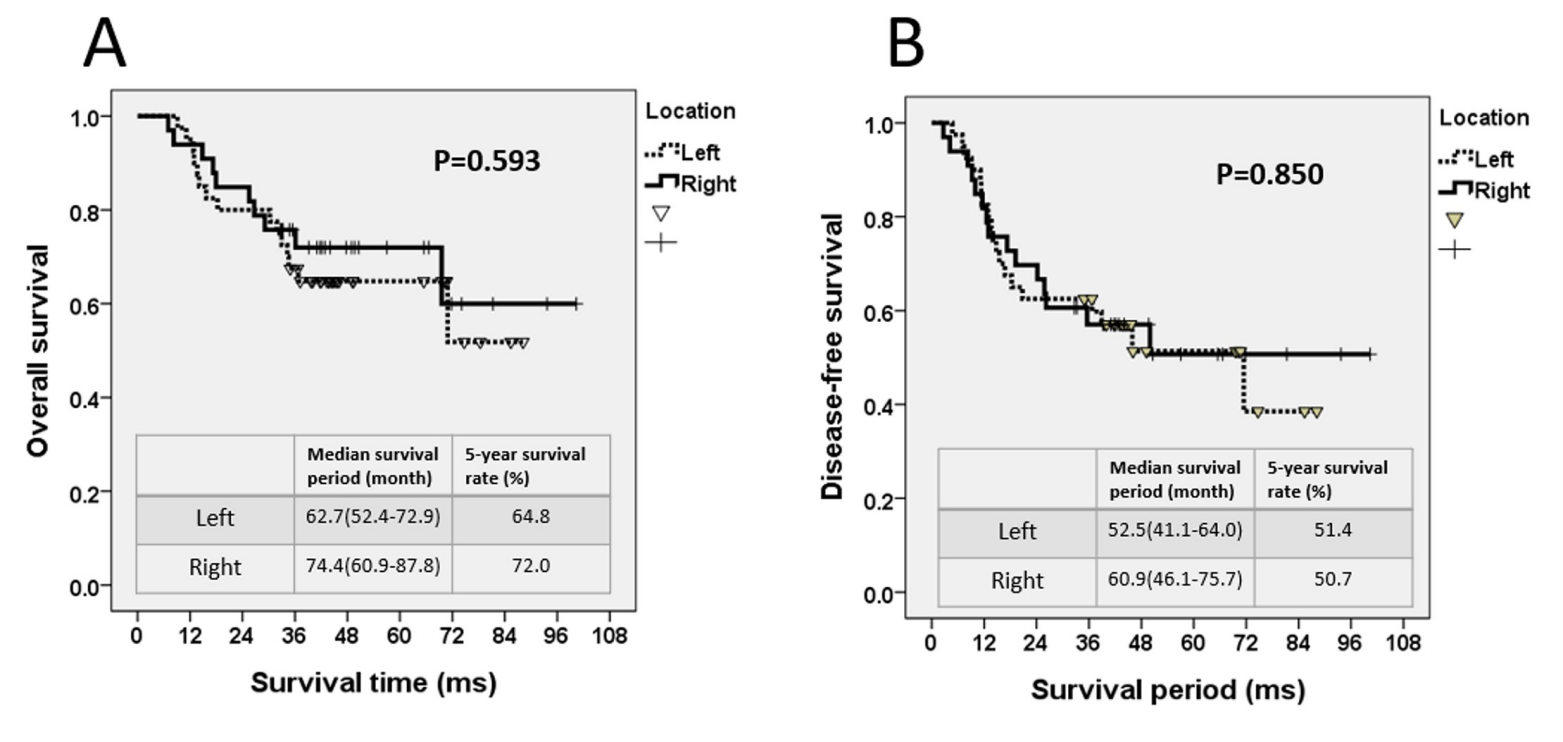
For the right and left side colon cancer, (A) the 5-year overall survival (OS) curve and (B) the 5-year disease-free survival (DFS) curve.

For patients with right-side colon cancer, the estimated 5-year OS rate was 72.0% with a median survival period of 74.4 months. Conversely, for patients with left-side colon cancer, the estimated 5-year OS rate was 64.8% with a median survival period of 62.7 months (*P* = 0.593, Fig 4A). The estimated 5-year DFS rate for right-side colon cancer was 50.7%, and the median DFS period was 60.9 months. Conversely, the estimated 5-year DFS rate for left-side colon cancer was 51.4%, and the median DFS period was 52.5 months (*P* = 0.850, Fig 4B).

## Discussion

With standard radical resection followed by adjuvant chemotherapy, the prognosis of LACC patients remains poor [11]. One of the reasons is that LACC involves challenges in radical tumor resection. Tumor invasion to other nearby organs or the lymph node around the root of the feeding artery results in increased complications and a positive resection margin [8]. Approximately one-third of LACC patients received multivisceral resection as initial treatment [6,22]; however, the R0 resection rate remained 40%–93% [11,23–25]. Some studies have stated that 13% of LACC patients failed to achieve R0 resection [6,26], and the 5-year OS rate was approximately 35%–47% [6,11,27]. Neoadjuvant chemotherapy has been administered to improve the outcome of LACC treatment because it can ensure chemosensitivity, improve the tumor response, and increase the R0 resection rate [6,7,11,28]. With the neoadjuvant strategy, the R0 resection rate of LACC can reach 84%–100% [6,7]. In this study, our rates of R0 resection, pCR, and OS were 95.8%, 17.3%, and 68.6%, respectively. Compared with multivisceral resection, neoadjuvant CCRT seems to be more feasible in LACC treatment. Many chemotherapy regimens have been introduced, with promising results, as preoperative therapy for LACC [6–8,11–13]. In this study, we included T3 tumors with extramural extension of >5 mm or T4 tumors diagnosed through imaging studies. Although the eligibility criterion of patients with LACC suitable for neoadjuvant CCRT remains debatable, the NCCN guidelines suggest that this treatment should be conducted for clinical T4b colon cancer patients [7,14,15,29].

In our present study, six patients (approximately 8%) failed to receive surgical resection after neoadjuvant CCRT. However, these tumors were considered unresectable after discussion in MDT cancer conferences. Adjacent organ invasion, including bladder, ureter, common iliac artery, and superior mesenteric vessels, was observed in these tumors. Furthermore, all patients received diversion stoma for bowel decompression. In other words, neoadjuvant CCRT did not delay treatment or induce unresectability. On the basis of our pathologic findings, one patient (1.4%) with progressed T stage, two patients (2.8%) with progressed N stage, and three patients (4.2%) with progressed TN stage. These findings indicate that a poor response or tumor progression are possible during neoadjuvant CCRT; nevertheless, surgical resection can still be performed as curative intent therapy. In our previous study, we observed that the overexpression of excision repair cross-complementing groups 1 and 2 is associated with poor responses to neoadjuvant CCRT [30]. However, with chemotherapy regimen modification, even T4b LACC can be converted from unresectable to resectable [30]. Obviously, an accurate CT scan image is essential in our treatment program. Many articles have described that CT is a reliable examination to assess the colon cancer stage and devise the subsequent surgical plan [7,11,12,16,19].

Many chemotherapy regimen protocols have been published. The FOxTROT trial enrolled 1052 patients from 85 centers in British, Denmark, and Sweden to assess the efficacy of neoadjuvant chemotherapy for LACC [7,11,28,31]. In this randomized controlled study, the neoadjuvant chemotherapy group presented fewer major surgical complications compared with the control group. Controls had an increased incidence of anastomosis leakage and intra-abdominal abscess and often needed additional surgery. Importantly, the control group was twice more likely to have a positive resection margin than the treatment group [7,28,31]. In a phase II trial, Jakobsen *et al*. assessed the efficacy of neoadjuvant capecitabine and oxaliplatin and concluded that neoadjuvant strategy is feasible and safe [16]. Similarly, Arredondo *et al*. investigated 65 LACC patients who received neoadjuvant FOLFOX. Four to six cycles of therapy were administered followed by surgical assessment and resection. The data suggest that neoadjuvant chemotherapy is safe and can accomplish major tumor regression [32]. In the current study, we adopted mFOLFOX6 as the chemotherapy regimen and repeated it biweekly until surgery based on our previous study results [11]. Overall, our results revealed a R0 resection rate of 95.8% and a pCR rate of 17.3%.

For the oncologic outcomes of patients with CRC, lymph node metastasis is a major poor prognostic factor. Many studies have proved that the lymph node metastatic ratio is an independent prognostic factor for survival in CRC patients [33–37]. In our previous study, neoadjuvant CCRT contributed to a high ypN0 rate of 82.4% and pN downstaging rate of 91.2% in LACC patients [11]. For patients with LARC, the neoadjuvant CCRT strategy led to the ypN0 rate of 78.9% and the pN downstaging rate of 56.8% [17,18]. Theoretically, these results indicate the eradication of lymph node metastasis through neoadjuvant CCRT [11,17,18]; however, no clear evidence exists regarding the relationship between patient survival and pathologic lymph node status. In this study, the ypN0 rate was 70.6%, and the pN downstaging rate was 80.0%. Our long-term follow-up results revealed that pN downstaging is an independent prognostic factor for patient OS. Furthermore, patients with pN downstaging had a significantly longer OS and DFS. Although patients with ypN0 cancer show a trend of longer OS and DFS, the result was not statistically significant.

Because neoadjuvant CCRT followed by radical resection has been widely applied for LARC treatment, pCR has been assumed to be a predictor of improved outcomes [38–42]. The pCR rate of LARC after neoadjuvant CCRT was approximately 9%–30% [39,41–44]. Compared with patients without pCR, patients with pCR have increased OS and DFS with a decreased incidence of LR [38–40,42,45,46]. Conversely, the role of pCR in LACC treatment remains inconclusive. Our previous study reported a pCR rate of 26.4% in patients with LACC who received neoadjuvant CCRT [11]. In a prospective observational study, Chang *et al*. investigated 60 LACC patients who underwent neoadjuvant CCRT and revealed a higher pCR rate in T3–T4a patients than in T4b patients (40.9% vs. 14.3%, *P* = 0.023) [6]. In our current results, 13 patients demonstrated a pCR rate of 17.3%. Only one patient with T4b cancer presented pCR, which is similar to the results of Chang *et al*. In univariate analysis in the present study, pCR exerted a significantly positive effect on patient OS; nevertheless, this finding did not reach statistical significance in multivariate analysis. Thus, the survival benefit from pCR may be affected by additional factors.

As per our results, patients with pCR presented increased OS with statistical significance, but not significantly increased DFS. We thought DM control rate was the main reason. Of 13 patients with pCR, four (30.7%) developed DM, which explains the findings regarding the DFS period. In Fig 3C, we can identify The DM control rate became nearly the same for patients with and without pCR in the fourth year. For patients in pCR group, the chemotherapy and target therapy can extend a longer OS than non-pCR group. This may explain the inconsistent results between our OS and DFS of pCR patients. On the other hand, a small patient number or shorter follow-up period might also affect our study results.

In our result, no LR was observed in patients with pCR. The finding of the excellent LR control rate in patients with pCR is similar with the results of LARC treatment. Jalilian *et al*. analyzed 127 patients with LARC who underwent neoadjuvant CCRT; their pCR rate was 14.96%, and 10.5% of patients with pCR developed DM in the follow-up period; however, none of the patients with pCR developed LR [41]. In a retrospective study, Smith *et al*. reported a pCR rate of 14.2% in 957 LARC patients who received neoadjuvant CCRT. No pelvic region recurrence or LR was observed [42]. Our data indicate that neoadjuvant CCRT has similar treatment effects on LACC and LARC. Basically, LACC patients with pCR have an excellent LR control rate but similar DM incidence compared with LACC patients without pCR.

Nevertheless, our patients with pCR presented significantly longer OS but nearly the same DM control rates compared with patients without pCR. In our previous study of neoadjuvant CCRT for T4 CRC, patients with pCR exhibited better OS and DFS but without statistical significance [47]. Jalilian *et al*. stated that patients with pCR had improved OS and DFS outcomes, but the results failed to reach statistical significance [41]. Furthermore, one meta-analysis conducted in 2012 reviewed the effect of neoadjuvant CCRT on LARC patients and found that patients with pCR had lower LR, lower DM, and increased OS and DFS periods, all with statistical significance [45]. In our opinion, neoadjuvant CCRT has similar therapeutic effects on both LACC and LARC. Moreover, pCR is a favorable factor for patients’ oncologic outcomes and disease control. Our contradictory data may have resulted from insufficient patient numbers. Therefore, a large database or multicenter study is warranted to investigate the relationship between pCR and treatment outcomes.

Based on toxicity and AEs, neoadjuvant therapy is considered safe and tolerable in most LACC patients. In a systemic review in 2020, Cheong *et al*. found that 12.5%–22% of LACC patients who underwent neoadjuvant chemotherapy developed granulocytopenia [19], and Zhou *et al*. reported that 4.3% and 21.7% of LACC patients who underwent neoadjuvant chemotherapy developed moderate to severe anemia and leukopenia, respectively [48]. Nevertheless, neoadjuvant radiotherapy was not applied in aforementioned studies. In our previous report, neoadjuvant CCRT for LACC was associated with 16.7% grade 3 anemia and 13.8% leukopenia [11]. Chang *et al*. reported that 20% of patients experienced moderate to severe myelosuppression after neoadjuvant CCRT; however, no details were provided regarding the subgroup incidence [6]. These data indicate that the incidence of severe hematologic AEs may be lower than our expectation even with CCRT. For non-hematologic AEs, the incidence of severe AEs was <10%. This result was consistent with our previous studies [6,11,19,48], and we suggested that neoadjuvant CCRT is a safe and tolerable treatment for patients with LACC.

According to our experience of LACC and LARC treatment, we supposed neoadjuvant CCRT followed by surgical resection is a safe and feasible strategy [11,18,49,50]. The pCR rate can reach 31.9% and the 3-year OS and DFS rate can be 89% and 75% respectively [49]. However, there are studies to discuss the benefit and possible overtreatment of neoadjuvant radiotherapy [50–52]. Schrag et al. described the strategy of neoadjuvant chemotherapy with selective rather than routine use of radiotherapy on clinical stage II and III rectal cancer [53]. The Preoperative Radiation or Selective Preoperative Radiation and Evaluation Before Chemotherapy and Total mesothelial excision (PROSPECT) trial also presented similar concept [51]. For low-risk rectal cancer, such as cT3N0, cT2N1, or cT3N1, selective use of pre-operative radiotherapy can be a treatment of choice [51]. In the past decade, although we achieved good treatment outcome by the strategy of neoadjuvant CCRT, there was no evidence to prove that routinely use of radiotherapy can provide additive benefit. More data and studies are necessary to adjust our current treatment program.

This study majorly reported the long-term survival outcomes of LACC patients who received neoadjuvant CCRT. Compared with most relevant articles, we presented the estimated 5-year rather than 3-year OS and DFS rates. Prognostic factors were analyzed based on the long-term follow-up period. Furthermore, the long-term oncologic outcome of patients with pCR was presented. However, this study still has some limitations. First, the number of patients was relatively small, with only 75 patients. Second, this was a retrospective study with an 8-year study period. On the basis of our experience with chemotherapy, we recently modified the treatment, particularly the postoperative adjuvant chemotherapy regimen. With increasing gene explorations, the treatment outcome can be further improved with the concept of precise medicine [54].

## Conclusion

Our study described that neoadjuvant CCRT followed by surgical resection can be a safe and feasible treatment strategy for LACC. Compared with directly multivisceral resection, neoadjuvant CCRT can be an option of initial treatment of LACC. Nevertheless, a large prospective, randomized control study may be needed before the introduction of CCRT in LACC in clinical practice.
